# Cardiac remodeling and subclinical myocardial dysfunction in patients with chronic kidney disease undergoing maintenance hemodialysis: a retrospective echocardiographic study

**DOI:** 10.3389/fcvm.2026.1948385

**Published:** 2026-09-25

**Authors:** Jiening Gao, Rong Liu, Zhaolin Yin, Yi Wang, Jingyu Chen, Meng Sun, Ruoling Han

**Affiliations:** Department of Ultrasound, The Fourth Hospital of Hebei Medical University, Shijiazhuang, Hebei, China

**Keywords:** cardiac remodeling, chronic renal failure, diastolic dysfunction, hemodialysis, left ventricular hypertrophy

## Abstract

**Background:**

Chronic kidney disease (CKD) is a major cardiovascular burden in patients undergoing maintenance hemodialysis and has traditionally been characterized by left ventricular hypertrophy (LVH). However, growing evidence suggests that myocardial dysfunction and mineral-metabolic abnormalities may be associated with cardiac remodeling beyond hypertrophy alone. This study aimed to comprehensively evaluate structural and functional cardiac alterations and their clinical-biochemical correlates in a retrospective cohort of long-term hemodialysis patients.

**Methods:**

We retrospectively identified 120 maintenance hemodialysis patients and 60 healthy controls. Comprehensive transthoracic echocardiography, including speckle-tracking, was analyzed to assess cardiac structure, myocardial mechanics, and diastolic function. Laboratory data, including hemoglobin and phosphate, were extracted. Correlation and multivariate regression analyses were performed to evaluate associations between clinical-biochemical variables and cardiac remodeling.

**Results:**

Hemodialysis patients showed significant cardiac remodeling, characterized by increased left ventricular mass index (LVMI), left atrial enlargement, and abnormal ventricular geometry. LVH was present in 68.3% of patients, with both concentric and eccentric hypertrophic patterns. Although Teichholz-derived left ventricular ejection fraction was relatively preserved, global longitudinal strain was significantly impaired and accompanied by a high prevalence of diastolic dysfunction. Dialysis duration, systolic blood pressure, phosphate, and hemoglobin were significantly associated with increased LVMI.

**Conclusions:**

Patients receiving maintenance hemodialysis exhibited a multidimensional pattern of cardiac remodeling involving structural abnormalities, subclinical myocardial dysfunction, and mineral-metabolic dysregulation. Within the hemodialysis cohort, greater LVMI was associated with longer dialysis vintage, higher systolic blood pressure and phosphate levels, and lower hemoglobin levels. The marked impairment in myocardial strain highlights the potential value of myocardial deformation imaging for characterizing cardiac dysfunction in this population. Integrated structural, functional, and biochemical evaluation may improve early cardiovascular risk stratification in CKD-related cardiomyopathy.

## 1 Introduction

For patients with chronic kidney disease (CKD), maintenance hemodialysis is life-sustaining and prolongs survival; however, this extended lifespan is accompanied by the progressive accumulation of cardiovascular structural and functional alterations (1). While dialysis effectively replaces renal clearance, it does not eliminate the profound cardiovascular burden imposed by the uremic milieu. Cardiovascular disease remains the dominant cause of mortality in this population, accounting for nearly 40%–50% of deaths worldwide (2, 3).

CKD-related cardiomyopathy is increasingly recognized as a heterogeneous clinical syndrome rather than a single structural entity, characterized by left ventricular hypertrophy, diastolic dysfunction, and subclinical systolic impairment. It is driven by hemodynamic overload, anemia, inflammation, and CKD-related mineral metabolism disorders, and is diagnosed based on integrated echocardiographic and biochemical features (4).

This raises a clinically relevant question: whether left ventricular hypertrophy alone is sufficient to capture the multidimensional nature of CKD-related cardiomyopathy (5). Traditionally, cardiac remodeling in end-stage renal disease has been interpreted as a hypertrophic response to chronic hypertension and increased afterload (6). However, this conventional view may underestimate the complexity of myocardial injury in patients undergoing long-term hemodialysis. Beyond pressure overload, the myocardium is continuously challenged by recurrent volume shifts, chronic inflammation, renal anemia, oxidative stress, and profound disturbances in mineral metabolism. These pathophysiological stressors collectively contribute to alterations in ventricular geometry, myocardial stiffness, and contractile efficiency. Such observations suggest that CKD-related cardiomyopathy should be viewed not simply as a disease of hypertrophy, but as a dynamic and multidimensional remodeling process.

Beyond hemodynamic overload, increasing evidence suggests that biological and metabolic disturbances play a central role in the progression of CKD-related cardiomyopathy. Abnormalities in mineral metabolism have emerged as important mediators linking chronic kidney disease to cardiovascular injury (7). Experimental and clinical studies indicate that persistent hyperphosphatemia and systemic inflammation are further associated with increased risk of myocardial fibrosis, ventricular stiffening, and impaired relaxation. However, how these biochemical abnormalities interact with structural remodeling and myocardial dysfunction in long-term hemodialysis remains incompletely understood.

Therefore, the present analysis was performed to quantify cardiac structural and functional alterations in maintenance hemodialysis patients and to explore their associations with clinical and biochemical parameters. By combining echocardiographic assessment and routinely obtained laboratory data, this study aims to provide a more integrated characterization of cardiac remodeling in chronic renal failure. Rather than inferring causality, we sought to evaluate whether cardiac remodeling could be conceptualized as a multidimensional process integrating hemodynamic stress, mineral metabolism disturbances, and myocardial functional impairment.

## 2 Materials and methods

### 2.1 Study design and population

This retrospective study was conducted at The Fourth Hospital of Hebei Medical University, Shijiazhuang, China between January 2022 and December 2024. A total of 120 patients receiving maintenance hemodialysis during the study period were identified from electronic medical records. Control participants were identified from the hospital health examination database covering the same study period. A total of 325 individuals who underwent routine health examinations and had complete clinical and echocardiographic records were initially screened. To obtain a control group with an age and sex distribution comparable to that of the hemodialysis cohort, these individuals were stratified by sex and 10-year age intervals, and random sampling was performed within each stratum. This process yielded 68 potential control candidates for detailed eligibility assessment. Eight individuals were subsequently excluded because of hypertension or diabetes mellitus (*n* = 4), abnormal renal function (*n* = 2), or poor echocardiographic image quality (*n* = 2). The remaining 60 individuals were included as healthy controls. The study protocol was approved by the Institutional Ethics Committee (approval number: 2024KY135) in accordance with the Declaration of Helsinki.

### 2.2 Inclusion and exclusion criteria

Inclusion criteria: Patients were eligible for analysis if they met all of the following criteria: (1) age ≥18 years; (2) chronic renal failure diagnosed according to the Kidney Disease: Improving Global Outcomes (KDIGO) clinical practice guidelines; (3) regular maintenance hemodialysis performed three times per week for at least 12 consecutive months; (4) stable clinical condition during the preceding 3 months, with no acute cardiovascular or infectious events requiring hospitalization and no changes in maintenance dialysis prescription; and (5) availability of complete clinical, laboratory, and echocardiographic data in the electronic medical records.

Exclusion criteria: Patients were excluded if they met any of the following criteria: (1) acute coronary syndrome within the previous 6 months; (2) severe valvular or congenital heart disease; (3) hypertrophic or dilated cardiomyopathy unrelated to chronic kidney disease; (4) persistent atrial fibrillation or other severe arrhythmias; (5) active infection, autoimmune disease, or malignancy; (6) severe liver dysfunction; (7) previous kidney transplantation or peritoneal dialysis; (8) inadequate echocardiographic image quality. Healthy controls were excluded if they had any of the following conditions: (1) chronic kidney disease; (2) established coronary artery disease, chronic heart failure, or atrial fibrillation; (3) hypertension; (4) diabetes mellitus; or (5) systemic inflammatory disorders.

### 2.3 Hemodialysis procedure

All patients had received routine bicarbonate-based hemodialysis using high-flux synthetic membranes as part of standard clinical care. Hemodialysis was performed three times per week, with each session lasting approximately 4 h. Blood flow rates were maintained between 250 and 300 mL/min, and dialysate flow rates were set at 500 mL/min. Ultrafiltration volume was individualized according to interdialytic weight gain, blood pressure, and clinical volume status. Dialysis adequacy was routinely monitored using single-pool Kt/V as part of standard clinical management, and all included patients had achieved the routine adequacy target (Kt/V ≥ 1.2) during the study period.

### 2.4 Clinical data collection

Demographic and clinical data were retrieved from electronic medical records and standardized patient records during the study period. The recorded variables included age, sex, body mass index (BMI), dialysis vintage, comorbid hypertension, diabetes mellitus, coronary artery disease (CAD), and blood pressure measurements. CAD was defined as a documented history of myocardial infarction, coronary revascularization, or angiographically confirmed stenosis ≥50% in at least one major epicardial coronary artery. Systolic blood pressure (SBP) and diastolic blood pressure (DBP) were measured before the midweek hemodialysis session after at least 10 min of rest in the seated position, and the average of three consecutive measurements was used for analysis. These blood pressure measurements therefore represented the pre-dialysis hemodynamic state.

### 2.5 Laboratory measurements

Fasting venous blood sample results were retrieved from electronic medical records and laboratory information systems. The analyzed laboratory parameters included hemoglobin, serum albumin, serum phosphate, and C-reactive protein (CRP). All laboratory analyses were performed in the Department of Laboratory Medicine of the hospital using standardized procedures.

### 2.6 Echocardiographic assessment

Comprehensive transthoracic echocardiography was performed using a Philips EPIQ 7C ultrasound system equipped with a 2.5–3.5 MHz phased-array transducer as part of routine clinical evaluation. All echocardiographic examinations were performed by two experienced physicians from the Department of Ultrasound Medicine using standardized imaging protocols. Echocardiographic assessment in hemodialysis patients was performed within 24 h after the midweek dialysis session as part of standard clinical practice. Because this was a retrospective study, the exact interval between completion of dialysis and echocardiographic examination and standardized documentation of post-dialysis dry-weight status were not consistently available for all patients.

Structural echocardiographic measurements included left ventricular end-diastolic diameter (LVEDD), interventricular septal thickness (IVST), left ventricular posterior wall thickness (LVPWT), left atrial diameter (LAD), and left atrial volume index (LAVI). Left ventricular mass (LVM) was calculated using the Devereux formula: LVM (g) = 0.8 × {1.04 × [(LVEDD + IVST + LVPWT) ^3^−LVEDD^3^]} + 0.6 Left ventricular mass index (LVMI) was calculated by indexing LVM to body surface area (BSA). Left ventricular hypertrophy (LVH) was defined as an LVMI >115 g/m^2^ in men and >95 g/m^2^ in women. Left ventricular geometric patterns were classified based on the combined assessment of relative wall thickness (RWT) and LVMI, including normal geometry, concentric remodeling, concentric hypertrophy, and eccentric hypertrophy (8).

### 2.7 Assessment of cardiac function

Left ventricular ejection fraction (LVEF) was derived from routine clinical echocardiographic records and had been calculated using the Teichholz method. Global longitudinal strain (GLS) was evaluated by two-dimensional speckle-tracking echocardiography based on three standard apical views, with values automatically calculated using dedicated analysis software. GLS was selected as a primary marker of subclinical systolic dysfunction due to its sensitivity to early myocardial injury in CKD populations.

Diastolic dysfunction was classified according to the recommendations of the American Society of Echocardiography and European Association of Cardiovascular Imaging (9). The evaluated parameters included mitral inflow E velocity, mitral inflow A velocity, E/A ratio, septal e′ velocity, lateral e′ velocity, and average E/e′ ratio. Based on these measurements, diastolic dysfunction was classified according to the current recommendations of the American Society of Echocardiography (ASE). In addition, left atrial reservoir strain (LASr) was measured using two-dimensional speckle-tracking echocardiography from the apical four-chamber view as a supplemental index of left atrial function and chronic filling pressure (10, 11). Tricuspid regurgitation peak velocity (TR velocity) was measured from the continuous-wave Doppler profile in the apical four-chamber view, when technically feasible. In patients with inadequate TR signal, grading was based on the remaining parameters (E/e′, septal e′, lateral e′, LAVI) according to the ASE algorithm.

### 2.8 Echocardiographic variables and study objectives

The primary outcome of the present study was the extent of cardiac remodeling, primarily assessed by LVMI. Secondary outcomes included the presence of LVH, impaired GLS, elevated E/e′ ratio, left atrial enlargement, and diastolic dysfunction. All outcomes were derived from retrospectively extracted echocardiographic and clinical data. To further describe the heterogeneity of cardiac remodeling in maintenance hemodialysis patients, the hemodialysis cohort was additionally stratified according to the presence of left ventricular hypertrophy. Patients were categorized into two subgroups: (1) patients without LVH and (2) patients with LVH according to sex-specific LVMI thresholds.

### 2.9 Reproducibility and quality control

To assess the reproducibility of echocardiographic measurements, the 120 hemodialysis examinations were assigned sequential identification numbers, and 20 examinations were selected using a computer-generated random-number procedure. These examinations were reanalyzed by the original observer and an independent second observer. Intraobserver and interobserver agreement for LVMI and GLS were evaluated using intraclass correlation coefficients (ICCs). All ICC values were greater than 0.85, indicating good measurement consistency and reliability. These analyses were performed based on echocardiographic data obtained from routine clinical examinations.

### 2.10 Statistical analysis

Statistical analyses were performed using IBM SPSS Statistics version 26.0 based on retrospectively extracted data. Continuous variables were expressed as mean ± standard deviation (SD) for normally distributed data or median (interquartile range) for non-normally distributed data, while categorical variables were presented as frequencies and percentages. Normality was assessed using the Kolmogorov–Smirnov test. Comparisons between groups were performed using the independent-samples t-test or Mann–Whitney U test for continuous variables, and the chi-square test or Fisher's exact test for categorical variables, as appropriate. Pearson correlation analysis was used to evaluate the associations between echocardiographic parameters and clinical-biochemical variables. Effect estimates were reported with 95% confidence intervals to indicate the precision of the observed associations. Because controls were selected by group-level matching rather than individual pairing, all between-group comparisons were performed using unpaired statistical tests. Multivariable linear regression analysis was performed with LVMI as the dependent variable. Covariates were selected *a priori* based on their clinical relevance and included age, sex, BMI, diabetes mellitus, history of coronary artery disease, dialysis vintage, systolic blood pressure, serum phosphate, and hemoglobin. All covariates were entered simultaneously into the model rather than selected according to bivariate statistical significance. Multicollinearity was assessed using variance inflation factors (VIFs). Standardized *β* coefficients with 95% confidence intervals and *P* values were reported. Model fit was summarized using R^2^, and the number of patients included in the model was reported.

## 3 Results

### 3.1 Baseline clinical characteristics

A total of 154 patients receiving maintenance hemodialysis were screened for eligibility, and 120 were included in the final analysis after predefined exclusions. Sixty healthy controls were included for comparison. The detailed participant selection process is shown in Figure 1. Demographic, clinical, and laboratory parameters were compared between the hemodialysis and control groups, as shown in Table 1. No significant differences were found in age, sex distribution, or BMI between the two groups (all *P* > 0.05). Systolic blood pressure, phosphate, and CRP were significantly elevated in hemodialysis patients, whereas hemoglobin and serum albumin levels were significantly reduced (all *P* < 0.001). These findings indicated substantial hemodynamic stress, chronic inflammation, mineral metabolism disturbance, and renal anemia in patients receiving long-term hemodialysis.

**Figure 1:**
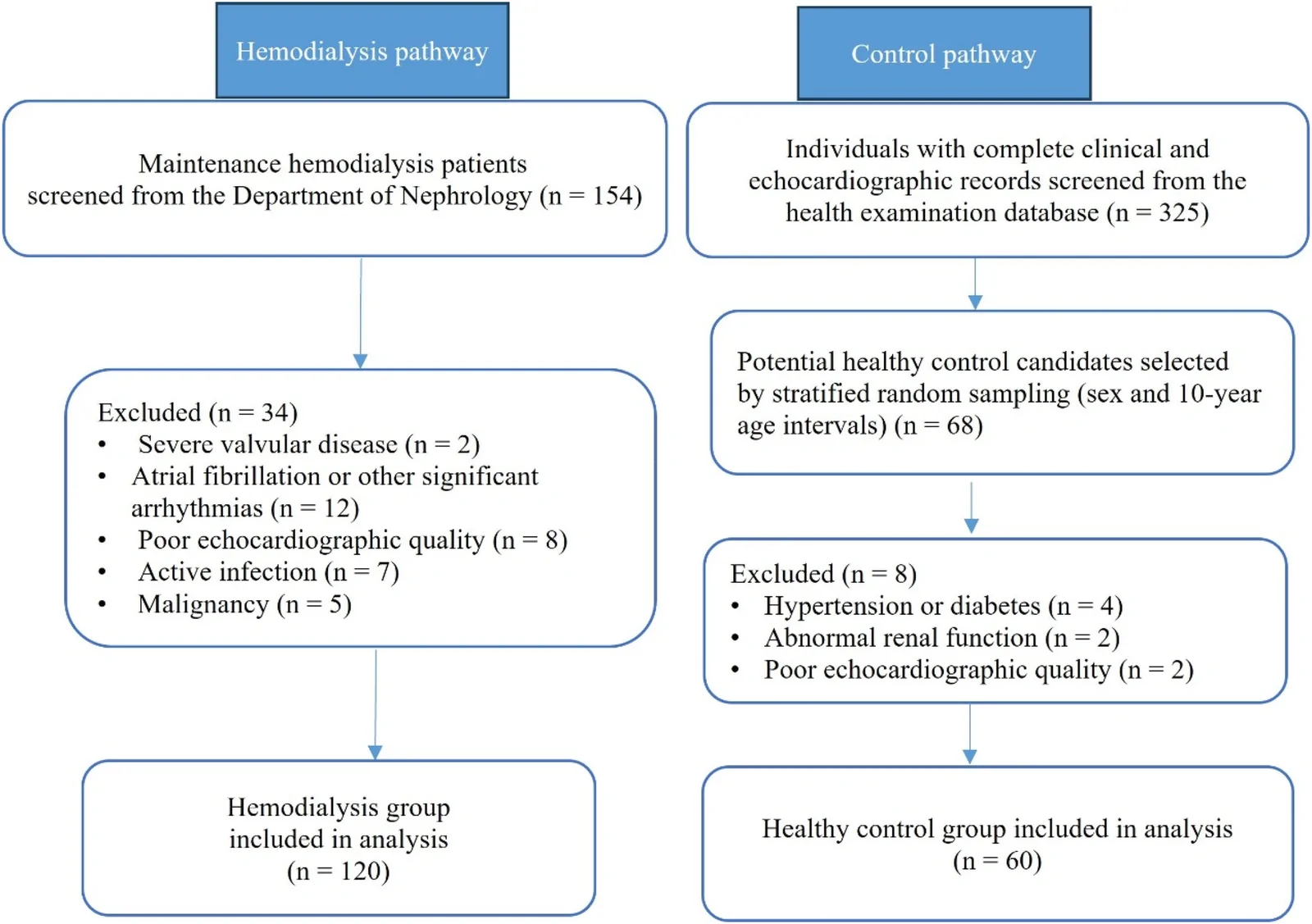
Flow diagram of participant selection and study inclusion.

**Table 1:** Baseline clinical characteristics of study participants.

Variable	Hemodialysis (*n* = 120)	Control (*n* = 60)	*P* value
Age (years)	56.8 ± 11.2	55.4 ± 10.7	0.42
Male sex, *n* (%)	82 (68.3%)	39 (65.0%)	0.81
BMI (kg/m^2^)	23.4 ± 3.8	24.1 ± 3.5	0.29
Dialysis vintage (years)	5.8 ± 2.9	—	—
Hypertension, *n* (%)	104 (86.7%)	0 (0%)	<0.001
Diabetes mellitus, *n* (%)	41 (34.2%)	0 (0%)	<0.001
Coronary artery disease, *n* (%)	28 (23.3%)	0 (0%)	<0.001
Systolic BP (mmHg)	148 ± 18	122 ± 11	<0.001
Hemoglobin (g/L)	98 ± 13	136 ± 11	<0.001
Albumin (g/L)	36.4 ± 4.1	43.7 ± 3.6	<0.001
Phosphate (mmol/L)	2.13 ± 0.51	1.18 ± 0.23	<0.001
CRP (mg/L)	7.8 ± 3.4	2.3 ± 1.1	<0.001

### 3.2 Echocardiographic structural parameters

To investigate structural cardiac differences between maintenance hemodialysis patients and healthy controls, echocardiographic structural parameters were analyzed retrospectively based on clinically obtained imaging data and are summarized in Table 2.

**Table 2:** Echocardiographic structural parameters.

Parameter	Hemodialysis	Control	*P* value
LVMI (g/m^2^)	145.6 ± 28.7	96.3 ± 14.2	<0.001
IVST (mm)	12.1 ± 1.8	9.4 ± 1.3	<0.001
LVPWT (mm)	11.7 ± 1.6	9.3 ± 1.1	<0.001
LVEDD (mm)	54.6 ± 6.1	48.7 ± 4.4	<0.001
RWT	0.43 ± 0.06	0.38 ± 0.04	<0.001
LAVI (mL/m^2^)	42.8 ± 8.4	26.1 ± 4.7	<0.001

Compared with healthy controls, maintenance hemodialysis patients exhibited significantly higher LVMI, IVST, LVPWT, LVEDD, RWT, and LAVI (all *P* < 0.001). As illustrated in Figures 2A–C, representative structural parameters (LVMI, IVST, and LAVI) showed marked differences between the two groups. To further characterize ventricular remodeling, left ventricular geometric patterns were assessed and are presented in Table 3.

**Figure 2:**
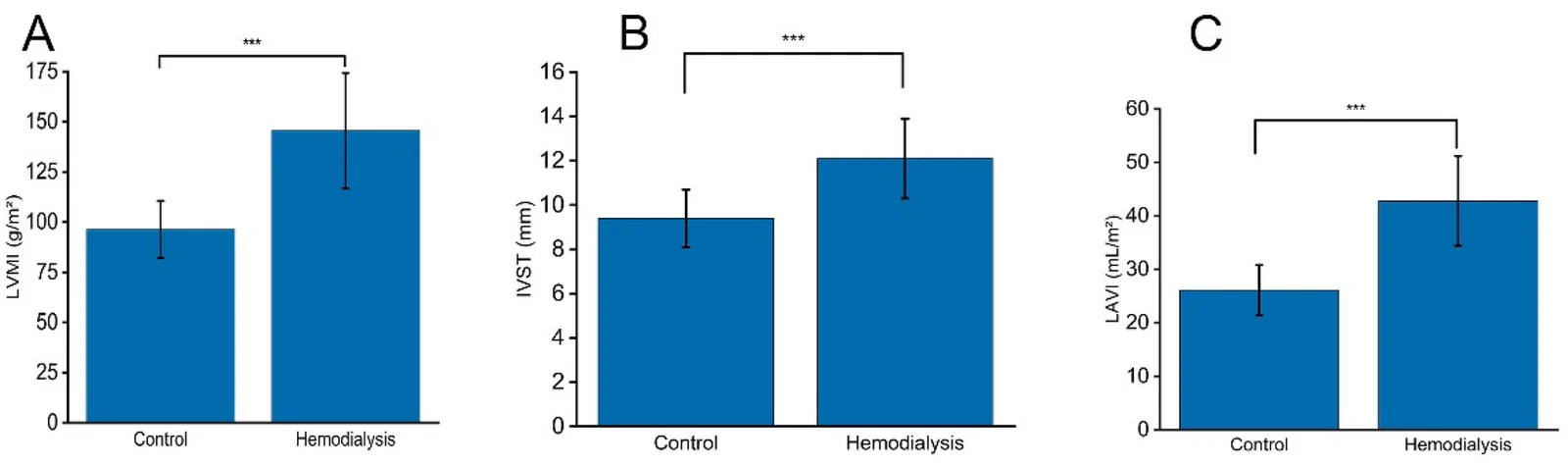
Structural cardiac remodeling in maintenance hemodialysis patients. **(A)** Left ventricular mass index (LVMI). **(B)** Interventricular septal thickness (IVST). **(C)** Left atrial volume index (LAVI).

**Table 3:** Distribution of left ventricular geometric patterns in hemodialysis patients.

Geometry Pattern	n (%)
Normal geometry	24 (20%)
Concentric remodeling	14 (11.7%)
Concentric hypertrophy	46 (38.3%)
Eccentric hypertrophy	36 (30.0%)
Total	120 (100%)
Total LVH	82 (68.3%)

As shown in Table 3, left ventricular hypertrophy was identified in 82 of 120 hemodialysis patients, corresponding to a prevalence of 68.3%. Among these patients, 46 exhibited concentric hypertrophy (38.3%), whereas 36 showed eccentric hypertrophy (30.0%). These findings indicate that adverse ventricular remodeling is common in patients undergoing long-term maintenance hemodialysis.

### 3.3 Echocardiographic functional parameters

To evaluate potential differences in cardiac function between maintenance hemodialysis patients and healthy controls, echocardiographic functional parameters were analyzed retrospectively based on clinically obtained echocardiographic data and are summarized in Table 4.

**Table 4:** Echocardiographic functional parameters.

Parameter	Hemodialysis	Control	*P* value
LVEF (%)	60.1 ± 7.2	61.8 ± 5.6	0.08
LASr (%)	22.4 ± 7.3	38.6 ± 6.5	<0.001
GLS (%)	−15.1 ± 2.8	−20.4 ± 2.3	<0.001
E/e′	16.3 ± 4.8	8.7 ± 2.1	<0.001
Septal e′ (cm/s)	5.8 ± 1.3	8.9 ± 1.6	<0.001
Lateral e′ (cm/s)	7.1 ± 1.7	11.2 ± 2.0	<0.001
TR velocity (m/s)	2.6 ± 0.5	2.1 ± 0.3	<0.001

For GLS, less negative values indicate worse myocardial deformation.

As shown in Table 4, left ventricular ejection fraction (LVEF) did not differ significantly between the two groups (*P* = 0.08). In contrast, global longitudinal strain (GLS) was significantly reduced and the E/e′ ratio was markedly increased in hemodialysis patients (both *P* < 0.001). Additionally, LA reservoir strain (LASr) was significantly lower in hemodialysis patients than in controls, and correlated inversely with E/e′ (r = −0.59, *P* < 0.001).

The functional alterations observed between the two groups are further illustrated in Figure 3. Hemodialysis patients exhibited a marked reduction in global longitudinal strain and elevation of the E/e′ ratio, indicating impaired myocardial deformation and diastolic performance in the presence of relatively preserved Teichholz-derived LVEF values. Consistently, diastolic dysfunction was identified in 87 of 120 patients (72.5%). Among patients with diastolic dysfunction, the distribution by grade was as follows: grade I (impaired relaxation) in 48 patients (55.2%), grade II (pseudonormal filling) in 28 patients (32.2%), and grade III (restrictive filling) in 11 patients (12.6%). TR velocity was measurable in 89 patients (74.2%); the remaining 31 patients were graded using the available echocardiographic parameters without the TR velocity component.

**Figure 3:**
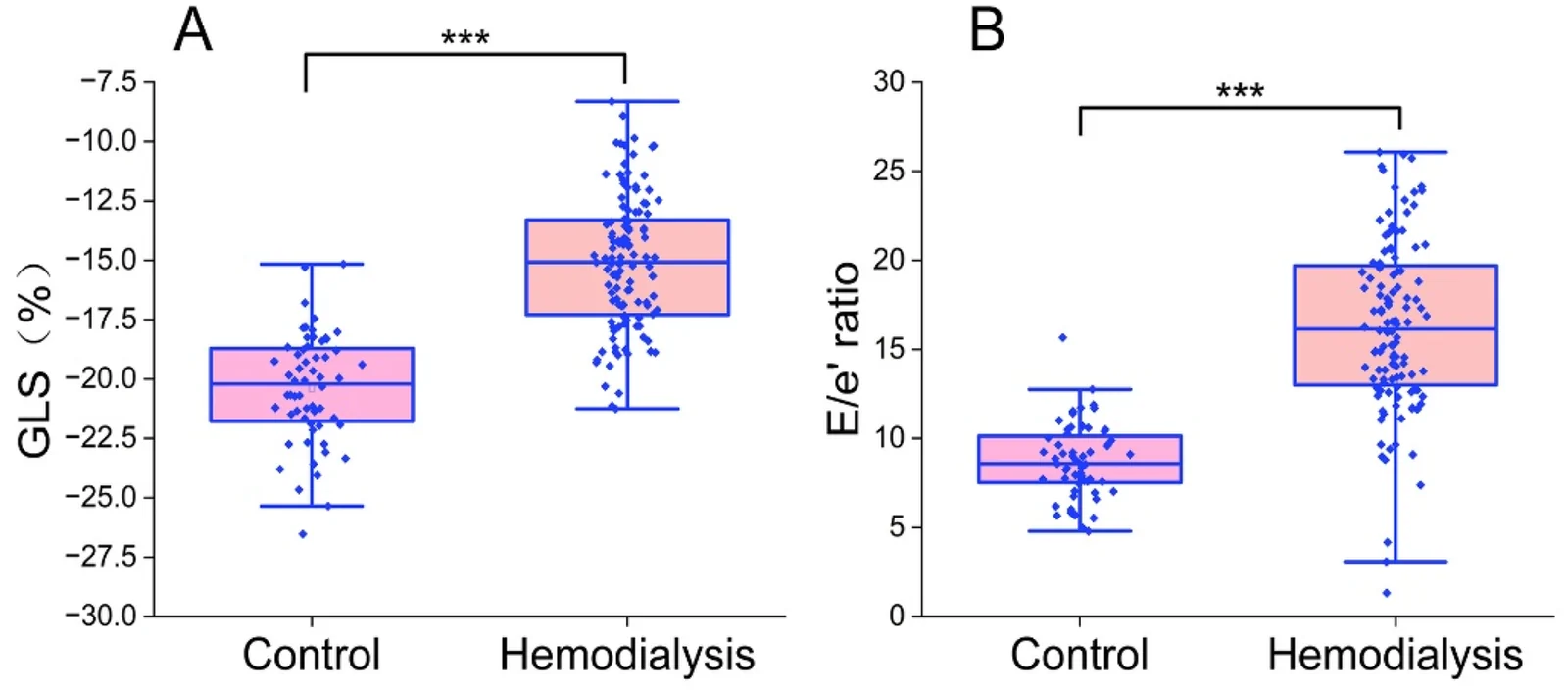
Echocardiographic assessment of myocardial function. **(A)** Global longitudinal strain (GLS). **(B)** E/e′ ratio. GLS is expressed as a negative percentage, with less negative values indicating worse myocardial deformation.

### 3.4 Stratified analysis according to left ventricular hypertrophy status in hemodialysis patients

Among the 120 patients receiving maintenance hemodialysis, 82 patients (68.3%) had LVH and 38 patients (31.7%) did not. Compared with patients without LVH, those with LVH exhibited greater left atrial enlargement and more pronounced myocardial functional impairment. Patients with LVH had higher LAVI, reduced GLS, and increased E/e′, together with a higher prevalence of diastolic dysfunction (Table 5). These findings indicate that LVH in maintenance hemodialysis patients is associated not only with structural remodeling but also with subclinical myocardial dysfunction.

**Table 5:** Comparison of cardiac remodeling and functional parameters between maintenance hemodialysis patients with and without left ventricular hypertrophy.

Parameter	Without LVH (*n* = 38)	With LVH (*n* = 82)	*P* value
Structural parameters			
LAVI (mL/m^2^)	36.9 ± 6.5	45.5 ± 8.2	<0.001
Functional parameters			
LVEF (%)	61.3 ± 6.8	59.6 ± 7.4	0.24
GLS (%)	−16.9 ± 2.5	−14.3 ± 2.7	<0.001
E/e′ ratio	13.1 ± 3.6	17.8 ± 4.7	<0.001
Diastolic dysfunction, *n* (%)	20 (52.6)	67 (81.7)	0.002

For GLS, less negative values indicate worse myocardial deformation.

### 3.5 Correlation analysis of cardiac remodeling

To explore the relationships between cardiac remodeling and clinical-biochemical parameters, correlation analyses were performed retrospectively based on clinically obtained echocardiographic and laboratory data and are summarized in Table 6. Representative correlations were selected for visualization based on their clinical relevance and pathophysiological significance and are illustrated in Figure 4. Specifically, LVMI showed significant correlations with dialysis duration (r = 0.56, *P* < 0.001) and serum phosphate (r = 0.43, *P* < 0.001). The E/e′ ratio was significantly correlated with serum phosphate concentration (r = 0.52, *P* < 0.001). Left atrial reservoir strain was also closely related to diastolic function, showing a significant inverse correlation with E/e′ (r = −0.59, *P* < 0.001). This association indicates that reduced atrial reservoir function accompanied greater impairment of left ventricular filling in the hemodialysis cohort.

**Table 6:** Correlation analysis between echocardiographic parameters and clinical-biochemical variables. E/e′ was analyzed in variables with established diastolic relevance.

Variable	LVMI, r	*P*-value	E/e′, r	*P*-value
Dialysis duration	0.56	<0.001	0.35	<0.001
Systolic blood pressure	0.47	<0.001	—	—
Serum phosphate	0.43	<0.001	0.52	<0.001
Hemoglobin	−0.38	<0.001	—	—
LASr	−0.41	<0.001	−0.59	<0.001

**Figure 4:**
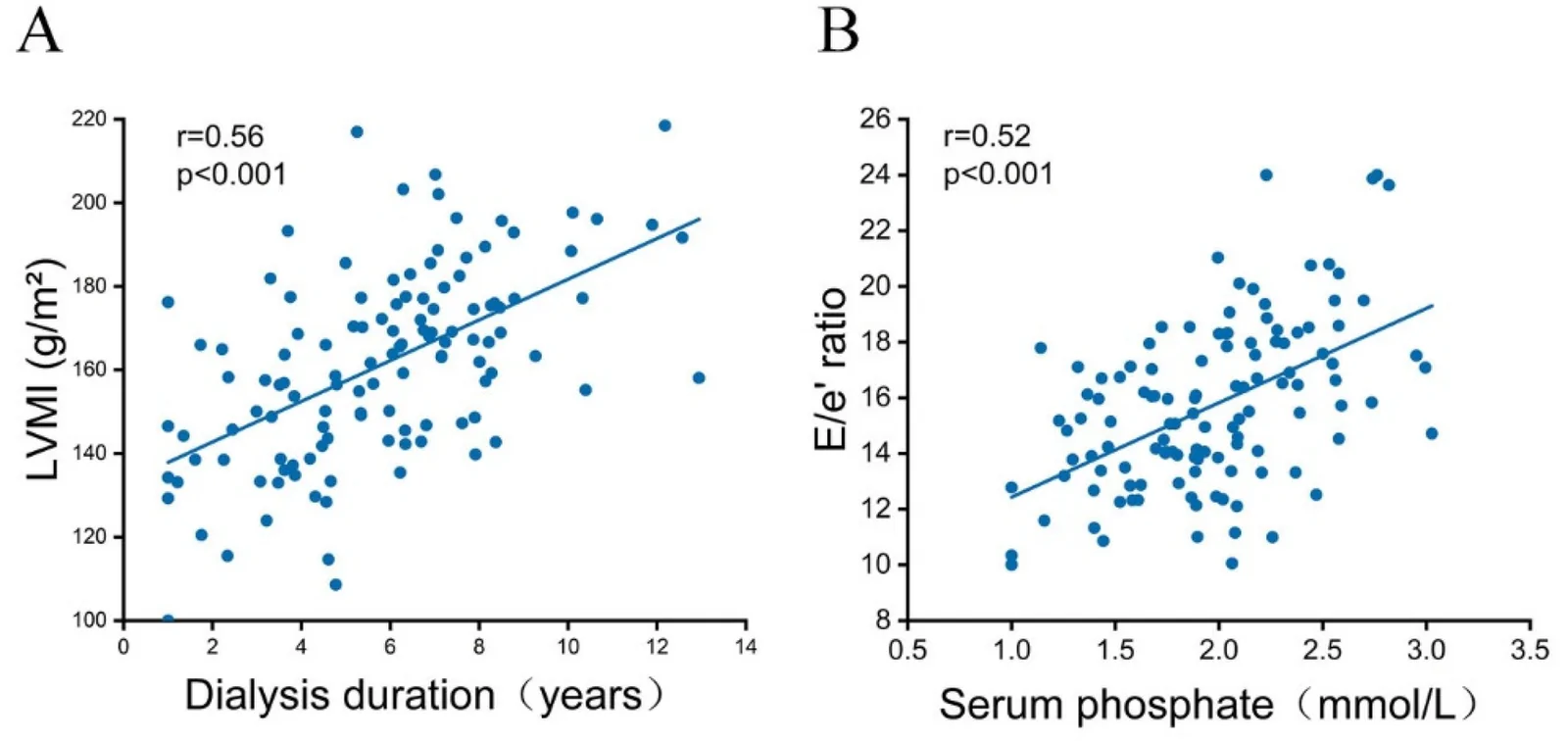
Correlation analysis of cardiac remodeling. **(A)** Correlation between dialysis duration and LVMI. **(B)** Correlation between serum phosphate and E/e′ ratio.

Overall, these findings indicate that cardiac structural and functional alterations in hemodialysis patients are associated with dialysis vintage, mineral metabolism disturbances, and anemia-related factors. These significant correlations provide the basis for subsequent multivariate regression analyses to further evaluate factors associated with cardiac remodeling.

### 3.6 Multivariable analysis of factors associated with LVMI

Multivariable linear regression analysis was performed with LVMI as the dependent variable, and the results are summarized in Table 7. After adjusting for age, sex, BMI, diabetes mellitus, and history of coronary artery disease, longer dialysis vintage, higher systolic blood pressure, and higher serum phosphate remained positively associated with LVMI, whereas higher hemoglobin was inversely associated with LVMI. The full model included 120 patients and explained 42% of the variance in LVMI (R^2^ = 0.42). These findings indicate that the associations of dialysis vintage, systolic blood pressure, serum phosphate, and hemoglobin with LVMI persisted after adjustment for relevant demographic and clinical factors.

**Table 7:** Multivariable linear regression analysis of factors associated with LVMI in patients receiving maintenance hemodialysis.

Variable	Standardized *β*	95% CI	*P* value
Age	0.08	−0.06–0.22	0.26
Male sex	0.06	−0.08–0.20	0.4
BMI	0.05	−0.09–0.19	0.48
Diabetes	0.11	−0.04–0.26	0.15
CAD history	0.1	−0.05–0.25	0.19
Dialysis vintage	0.41	0.28–0.55	<0.001
Systolic blood pressure	0.32	0.17–0.47	<0.001
Serum phosphate	0.29	0.11–0.46	0.002
Hemoglobin	−0.27	−0.41 to −0.13	0.001

β coefficients are standardized. Model *n* = 120; R^2^ = 0.42. BMI, body mass index; CAD, coronary artery disease; LVMI, left ventricular mass index.

## 4 Discussion

### 4.1 Principal findings and integrated interpretation

This retrospective study provides an integrated characterization of cardiac structural and functional abnormalities in patients with chronic kidney disease undergoing maintenance hemodialysis. Compared with healthy controls, hemodialysis patients exhibited greater left ventricular mass, a higher prevalence of abnormal ventricular geometry, left atrial enlargement, impaired global longitudinal strain, and more pronounced diastolic dysfunction. These between-group comparisons were interpreted descriptively because differences in accompanying cardiovascular risk factors between the groups preclude attribution of these abnormalities specifically to dialysis or uraemia.

Within the hemodialysis cohort, patients with LVH showed greater left atrial enlargement, more impaired global longitudinal strain, higher E/e′ ratios, and a higher prevalence of diastolic dysfunction than those without LVH. Multivariable analysis further showed that longer dialysis vintage, higher systolic blood pressure, and higher serum phosphate were positively associated with LVMI, whereas higher hemoglobin was inversely associated with LVMI after adjustment for relevant demographic and clinical factors. Taken together, these findings provide an integrated characterization of structural remodeling, myocardial functional impairment, and associated clinical-biochemical factors within the same hemodialysis cohort. This integrated assessment may provide a more comprehensive characterization of cardiac involvement in patients receiving maintenance hemodialysis. Importantly, the observed between-group echocardiographic differences should be interpreted in the context of substantial differences in cardiovascular risk factors. Hypertension, diabetes mellitus, and coronary artery disease were present in the hemodialysis cohort but absent in the healthy control group, and these conditions may independently contribute to increased LVMI, altered ventricular geometry, and impaired myocardial and diastolic function. Because the between-group echocardiographic comparisons were not adjusted for these potential confounders, they are descriptive and should not be interpreted as effects specifically attributable to hemodialysis or uremia.

### 4.2 Cardiac remodeling extends beyond left ventricular hypertrophy

Left ventricular hypertrophy was highly prevalent in patients with chronic kidney disease receiving maintenance hemodialysis, but the pattern of remodeling was not uniform (12). Both concentric and eccentric hypertrophic phenotypes were observed, indicating that cardiac adaptation in chronic kidney disease is shaped by multiple and potentially competing stressors (13–15). The mean RWT in the hemodialysis cohort (0.43) was close to the 0.42 threshold used for geometric classification. Therefore, the distinction between concentric and eccentric patterns may be sensitive to small variations in echocardiographic measurements and should be interpreted cautiously. Pressure overload related to hypertension may preferentially be associated with concentric remodeling, whereas chronic volume shifts and anemia may be associated with chamber enlargement and eccentric hypertrophy. The concomitant increase in left atrial volume further suggests chronically elevated filling pressure and sustained diastolic burden. These findings underscore that ventricular hypertrophy alone is insufficient to capture the complexity of cardiac remodeling in hemodialysis patients, and that geometric phenotyping may provide additional information regarding disease heterogeneity and cardiovascular vulnerability. This concept is consistent with the view that CKD-related cardiomyopathy is not merely a disease of hypertrophy, but a dynamic and multidimensional process. Our subgroup analysis further showed that patients with LVH had significantly greater left atrial enlargement, more impaired GLS, and higher E/e′ ratios than those without LVH. These findings indicate that LVH in maintenance hemodialysis is associated with both structural remodeling and subclinical myocardial dysfunction rather than representing an isolated increase in myocardial mass.

### 4.3 Myocardial mechanical impairment assessed by global longitudinal strain

A key observation of this study was the marked impairment in global longitudinal strain among patients receiving maintenance hemodialysis (16). Although Teichholz-derived LVEF values were relatively preserved, the methodological limitations of this approach in patients with altered ventricular geometry should be considered when interpreting the relationship between LVEF and GLS. Reduced GLS may nevertheless provide complementary information regarding myocardial mechanical impairment that is not fully captured by conventional LVEF assessment. Coronary artery disease should also be considered when interpreting reduced GLS, as underlying ischemic heart disease may contribute to impaired myocardial deformation and cannot be fully separated from kidney disease-related cardiac remodeling in this retrospective cohort. Left atrial reservoir strain was also markedly reduced in the hemodialysis cohort and was inversely correlated with E/e′. This finding suggests that atrial functional impairment accompanies the broader pattern of myocardial and diastolic abnormalities observed in these patients. Longitudinal myocardial fibers, which are predominantly located in the subendocardial region, are particularly vulnerable to microvascular dysfunction, fibrosis, metabolic stress, and pressure–volume fluctuations. Therefore, reduced global longitudinal strain may represent an early marker of myocardial injury before overt systolic failure becomes apparent (17). The simultaneous elevation of E/e′ and high prevalence of diastolic dysfunction further indicate substantial impairment of diastolic function in this population. The reduced LASr and its correlation with E/e′ suggest that LA dysfunction reflects not only elevated filling pressure but also intrinsic atrial myopathy, indicating that diastolic impairment in CKD involves active myocardial deformation beyond passive hemodynamic changes (18). The control LASr value in this study was near the lower boundary of commonly reported reference ranges. However, LASr values may vary according to imaging platform, analysis software, and measurement methodology, and comparisons with published reference values should therefore be interpreted with caution (19, 20). These results are consistent with subclinical myocardial dysfunction in long-term hemodialysis patients and highlight the value of myocardial deformation imaging for detecting early cardiac impairment.

### 4.4 Potential mechanisms linking dialysis exposure, mineral metabolism, and myocardial injury

In this study, LVMI was associated with dialysis vintage, systolic blood pressure, phosphate, and hemoglobin within the hemodialysis cohort; however, these observational associations should not be interpreted as evidence of causal pathways. Longer dialysis vintage was positively associated with LVMI in the present cohort. However, this association should not be interpreted as evidence of a cumulative effect of dialysis, because the inclusion of prevalent hemodialysis patients may have introduced survivor selection. Longitudinal studies beginning at dialysis initiation are needed to clarify the temporal relationship between dialysis duration and cardiac remodeling. In addition, systolic blood pressure was measured before dialysis, whereas echocardiography was performed after dialysis; therefore, the observed association between SBP and LVMI should be interpreted as an association with pre-dialysis blood pressure rather than with the hemodynamic state at the time of echocardiography. In parallel, phosphate retention may be associated with cardiomyocyte hypertrophy, interstitial fibrosis, and impaired myocardial relaxation. Anemia may further aggravate remodeling by increasing cardiac output demand and reducing myocardial oxygen delivery (21). Although the observational design does not establish causality, these findings are consistent with a framework in which CKD–mineral and bone disorder, anemia–inflammation burden, and hemodynamic overload interact and are associated with an increased risk of progressive myocardial remodeling and mechanical dysfunction. Based on these observed associations, Figure 5 presents a hypothesis-generating framework integrating clinical-biochemical factors with structural and functional cardiac abnormalities in patients receiving maintenance hemodialysis.

**Figure 5:**
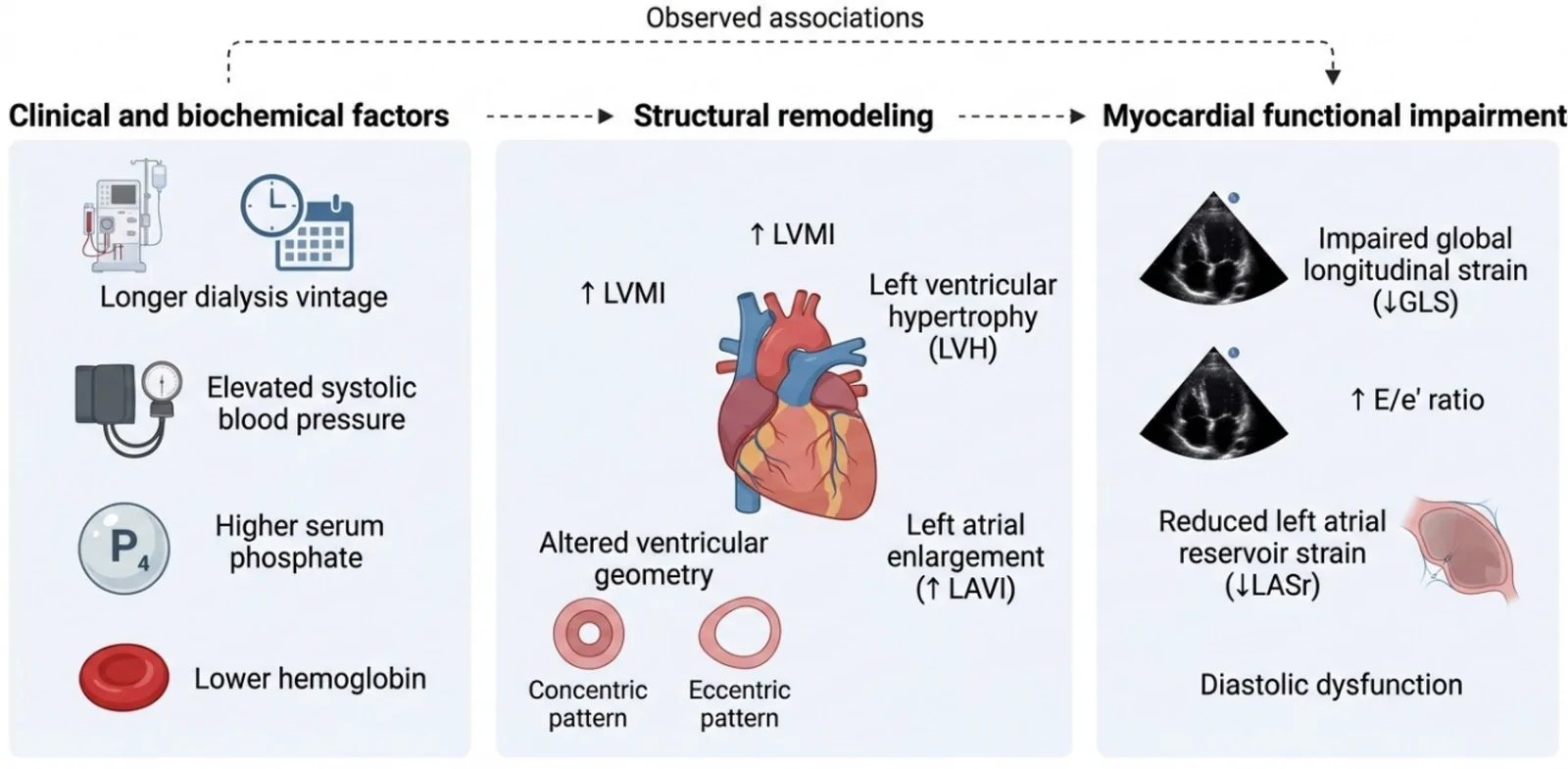
Descriptive framework of observed clinical-biochemical and echocardiographic associations in maintenance hemodialysis. The framework integrates the observed associations among clinical-biochemical factors, structural cardiac remodeling, and myocardial functional abnormalities in the present cohort. The proposed relationships are hypothesis-generating and should not be interpreted as established causal pathways.

### 4.5 Clinical implications, limitations, and future perspectives

These findings have several clinical implications. First, myocardial deformation imaging may provide complementary information for characterizing myocardial mechanical impairment in hemodialysis patients beyond conventional echocardiographic assessment. Second, combining structural indices, myocardial strain, diastolic parameters, and biochemical markers may improve early risk stratification. Third, abnormalities in phosphate and hemoglobin may represent potentially modifiable or monitorable factors associated with adverse cardiac remodeling (22). This retrospective study suggests that integrated structural, functional, and biochemical evaluation may provide a more comprehensive approach for cardiovascular risk assessment in this population. Several limitations should be acknowledged, including the retrospective, single-center design and reliance on echocardiographic rather than tissue-level characterization of myocardial fibrosis. In addition, LVEF was derived using the Teichholz method from routine clinical echocardiographic records. Because the imaging data required for reliable retrospective reanalysis using the biplane Simpson's or 3D method were not available for all participants, the interpretation of LVEF, particularly in patients with altered ventricular geometry, should be made with caution. Because healthy controls were selected to exclude major cardiovascular risk factors, including hypertension and diabetes mellitus, whereas these conditions were common in the hemodialysis cohort, residual and unmeasured confounding is a major limitation of the between-group comparisons. These comparisons were not adjusted for cardiovascular risk factors and should therefore be interpreted as descriptive; they cannot distinguish dialysis- or uremia-related abnormalities from those associated with the differing cardiovascular risk profiles of the two groups. The timing of echocardiography within 24 h after dialysis also allowed variation in post-dialysis loading conditions, and exact examination intervals and dry-weight status were not consistently available. This may have influenced volume-sensitive echocardiographic measurements, including LV dimensions, LAVI, E/e′, and atrial strain. In addition, the inclusion of prevalent hemodialysis patients may have introduced survivor selection, particularly when interpreting associations between dialysis vintage and cardiac remodeling. Future multicenter longitudinal studies integrating cardiac magnetic resonance, circulating fibrosis markers, and metabolic profiling may help clarify temporal relationships and identify potential therapeutic windows for CKD-related cardiomyopathy progression.

## 5 Conclusions

In conclusion, patients receiving maintenance hemodialysis exhibited multidimensional cardiac abnormalities involving structural remodeling, impaired myocardial mechanics, and metabolic disturbances. Within the hemodialysis cohort, dialysis vintage, systolic blood pressure, phosphate, and hemoglobin were associated with LVMI, supporting the multifactorial nature of cardiac remodeling in this population.

## Data Availability

The raw data supporting the conclusions of this article will be made available by the authors, without undue reservation.
